# Fentanyl exposure during preconception and gestation permanently dysregulates endogenous opioid peptides and sympathoadrenal-medullary axis in the offspring

**DOI:** 10.1042/CS20256962

**Published:** 2025-11-06

**Authors:** Nermin Ahmed, Carolina Dalmasso, Navid S. Tavakoli, Pedro Peñalver Abed, Meghan B. Turner, Lindsay C. Czuba, Ricardo M. Pautassi, Pavel I. Ortinski, Analia S. Loria

**Affiliations:** 1 Department of Pharmacology and Nutritional Sciences, College of Medicine; 2 Department of Neuroscience, College of Medicine; 3 Instituto de Investigaciones Médicas M. y M. Ferreyra (INIMEC-CONICET-UNC) Universidad Nacional de Córdoba, Argentina; 4 Department of Pharmaceutical Sciences, College of Pharmacy; 5 SAHA Cardiovascular Center, University of Kentucky Lexington, KY, 40536

**Keywords:** endogenous opioid peptides, opioid withdrawal, preconception, prenatal fentanyl exposure, self-administration

## Abstract

In the United States, the alarming increase in opioid use disorder diagnoses during pregnancy in the last decade has increased the incidence of neonatal opioid withdrawal syndrome (NOWS). Although 8 per 1,000 newborns are diagnosed with NOWS each year, the lack of prospective studies is a roadblock in the development of approaches to reduce adverse health outcomes in this vulnerable population. This study used a preclinical model to assess short- and long-term effects of preconceptional and gestational fentanyl (FEN) exposure on morphometrics, hormonal plasma profile, and sensitivity to opioid re-exposure in the offspring. Sprague Dawley female rats self-administered FEN citrate [fixed-ratio 1 (FR1), 2.5 μg/kg] or vehicle (NaCl 0.9%) during preconception and until gestational day 21. *In utero* fentanyl exposure (IUFE) did not influence neonatal weight and morphometrics; however, IUFE pups exhibited a higher frequency of behaviors indicative of somatic withdrawal compared with controls (CTLs). In male and female weanlings, IUFE induced the dysregulation of endogenous opioid peptides (EOPs) and increased metanephrine levels compared with CTL counterparts. However, only adult females with IUFE showed increased EOPs and metanephrine levels, FEN-induced hyperalgesia, and greater FEN-induced hypotensive and bradycardic effects compared with CTL counterparts. This preclinical model suggests a long-lasting association between IUFE-induced neuroendocrine dysregulation and adverse effects of opioid re-exposure in female offspring.

## Introduction

The opioid crisis is one of the greatest public health threats in the United States. In 2023, adverse health outcomes due to opioid use disorder (OUD) and opioid misuse, principally driven by illicit fentanyl (FEN), generated a total economic burden of $2.7 trillion [1–3]. While a national decline that was reported in 2023 and 2024 is encouraging news, overdose remains the leading cause of death for Americans aged 18–44, [4] highlighting the importance of sustaining preventive efforts. The last decade has seen an exponential increase in diagnoses of OUD during pregnancy [5], which was associated with a five-fold increase in neonatal opioid withdrawal syndrome (NOWS) incidence [5–7].

FEN is a strong analgesic widely used to relieve acute and chronic pain via the activation of the mu-opioid receptor (μ-OR) [8]. FEN has a high misuse liability, and its continued use can lead to opioid dependence, a condition in which the sudden reduction in drug intake triggers a surge of central nervous system overactivity, driven by increased norepinephrine release [9,10]. This neurotransmitter imbalance results in a range of intense withdrawal symptoms, including severe cravings and heightened pain sensitivity [11]. Neonates whose mothers abruptly ceased or reduced FEN use at the time of pregnancy are also likely to suffer withdrawal symptoms. Specifically, NOWS is characterized by central nervous system hyperexcitability, tremors, irritability, high-pitched crying, tight muscle tone, hyperactive reflexes, and seizures [7,11–13], [14,15].

Somatic withdrawal varies in severity and duration from a few days to weeks; however, heightened pain sensitivity can persist for months or even years after the discontinuation of opioid use in humans [16,17], as well as in rodents [18]. Current approaches to treat withdrawal in adults and newborns involve partial µ-OR agonists (e.g. buprenorphine) [19–22] or sympatholytic drugs such as α2-adrenergic receptor type agonists (e.g. clonidine) [23,24]. The use of other µ-OR antagonists (e.g., naloxone), which are very effective in adults, is avoided in newborns due to the greater risk of cardiorespiratory depression. Consequently, preclinical models are key to identifying new therapeutic targets that reduce the risk of chronic disease in disadvantaged populations.

An important contribution of preclinical models of maternal OUD is to dissect the role of different components of the endogenous opioid system. This comprises endogenous opioid peptides (EOPs), including pro-enkephalins, proopiomelanocortin, and pro-dynorphin, and their derived peptides, and three OR subtypes expressed in most tissues: µ-OR, kappa OR (κ-OR), and delta OR [25]. ORs are expressed in the placenta and the brain of neonates in gestational weeks 12–13, where EOPs also modulate brain development [26,27]. Physiologically, maternal β-endorphin plasma levels increase during pregnancy from week 6 (20 fmol/ml) to delivery (>120  fmol/ml) [28]. By postnatal day 5 (PD5), EOP levels in neonates and mothers decrease to physiological levels [28]. However, β-endorphin levels remain elevated in opioid-exposed infants 40 days after birth [28] and continue to increase about 100-fold compared with young age-matched controls (CTLs) [28,29].

The activation of the hypothalamic–pituitary–adrenal (HPA) axis and the sympathetic–adrenomedullary system in response to stressors leads to increased EOPs, as well as synthesis and release of glucocorticoids and catecholamines from the adrenal cortex and medulla [30]. Prenatal opioid exposure can disrupt the HPA axis development in the fetus, leading to persistent changes in stress response, brain structure, and behavior in the offspring. This can manifest as an altered stress response, changes in endocannabinoid signaling, and other neurodevelopmental effects [31]. Nevertheless, how transplacental FEN may permanently influence the production of neuroendocrine factors remains unclear.

The lasting effects of *in utero* fentanyl exposure (IUFE) on opioid responsivity in the offspring remain unclear. Thus, this study exposed female rats to fentanyl self-administration (FEN-SA) during preconception and pregnancy to investigate the effects of IUFE on the offspring’s birth weight, morphometrics, litter size, and somatic signs of withdrawal. In addition, we determined the growth trajectory and plasma EOPs profile and other adrenal-derived hormones. We also re-exposed a subset of adult offspring to analgesic doses of FEN to evaluate the lasting effects of IUFE on thermal pain, pharmacokinetics, blood pressure, and heart rate (HR).

## Materials and methods

### Animals

The study, which followed ARRIVE 2.0 guidelines and those of the National Institutes of Health Guide for the Care and Use of Laboratory Animals, was performed at the University of Kentucky, under the Institutional Animal Care and Use Committee under protocol number 2020–3477. Fourteen-week-old Sprague Dawley female and male rats were used for breeding, had *ad libitum* access to food and water, and were housed in a pathogen-free environment with constant temperature and humidity, with a 12:12 h light/dark cycle. The animals had *ad libitum* access to water and a regular chow diet [24% protein, 58% carbohydrates, and 18% fat with 3.1 kcal/g gross energy (Teklad 8604, Madison, WI)] and were housed in the same conditions.

### Experimental design

**Figure 1:**
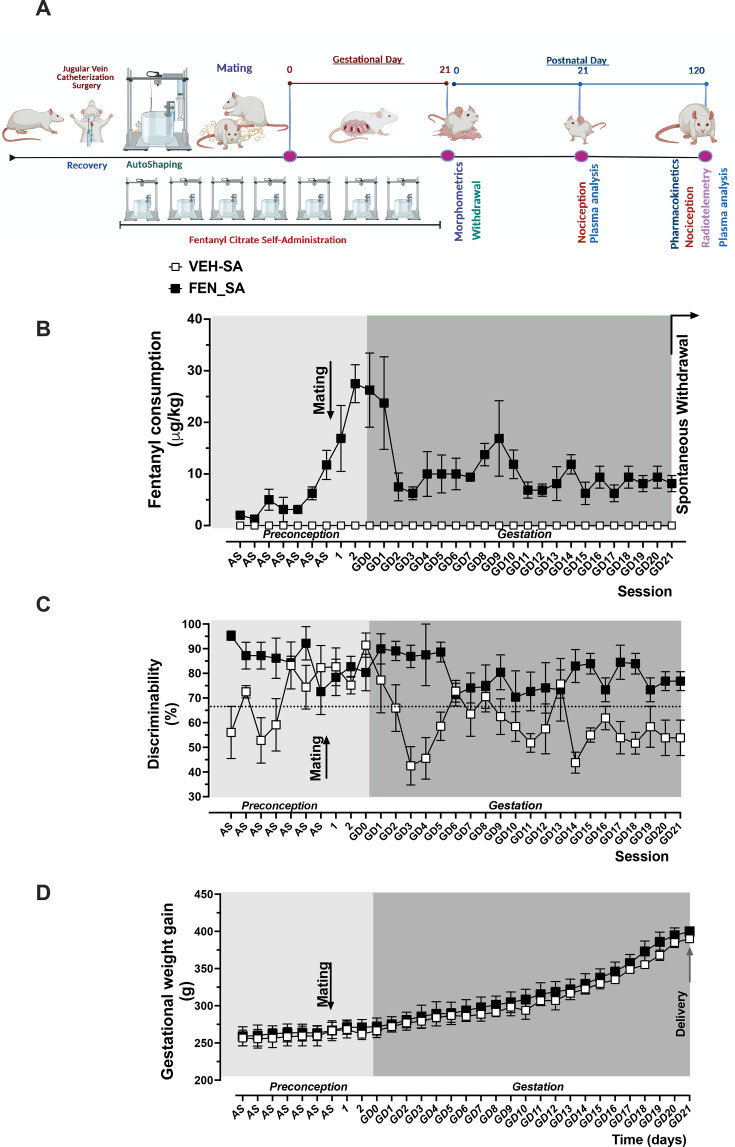
Fentanyl self-administration in pregnant rats (**A**) timeline of events involving dams and their offspring, (**B**) fentanyl consumption in female rats self-administering VEH or FEN during preconception and gestation, (**C**) discrimination percentage of active versus inactive lever presses in female rats self-administering vehicle or fentanyl; the horizontal dotted line represents 66.6% active lever press discrimination passing criteria during preconception and gestation, and (**D**) weight gain projection during preconception and gestation. Data were collected, analyzed, averaged per group, and reported as mean ± SEM; *n*=3 dams per group.µg=microgram; kg=kilogram; g=gram. *P<0.05 versus control. FEN, fentanyl; SA, self-administration; VEH, vehicle; D, gestational day.

A vaginal smear was performed between 8:00 and 9:00 AM on cycling female rats to establish timed pregnancy. After one week, dams were implanted with a chronic jugular vein catheter, and vaginal smears continued through recovery. Then, rats were trained to self-administer FEN or vehicle (VEH, sterile NaCl 0.9%). After seven days of training, the females were mated on the proestrus phase of the hormonal cycle. The presence of a sperm plug determined gestational day 0. Males were then removed, and females continued the self-administration sessions while monitoring weight gain daily until delivery Maternal weight gain during preconception and gestation was assessed daily. Body weight was assessed in all the pups at birth and was recorded weekly until the endpoints, PD21 or PD120. Newborn offspring were marked for identification, and morphometric measurements were taken. After culling pups to 10–12 per litter via decapitation, ten-min videos were recorded simultaneously in each litter at PD0, 1, and 3, placing the offspring in a plexiglass container to assess somatic signs of withdrawal. A subset of rats was subjected to a hot plate test at PD21. At PD120, a hot plate test was performed on a subset of female rats to determine nociception, another subset to determine FEN plasma levels, and a third subset of rats was implanted with radiotelemetry at PD90. After a ten-day recovery period, blood pressure and HR were assessed at baseline and in response to a sedative dose of FEN (20 and 200 µg/kg, s.c., respectively), allowing a one week recovery between each measurement. At each timepoint, euthanasia was performed by anesthetizing the rats with ketamine/xylazine (100/10 mg/kg, IP) and conducting a thoracotomy for blood collection via cardiac puncture. Blood was centrifuged (3000 rpm, 10 min, 4°C), and plasma was separated, snap frozen, and stored at −80°C until analysis. The overall experimental design is depicted in Figure 1A.

### Randomization of the offspring

In each litter, all offspring (6–8 males and 5–7 females) were subjected to morphometrics, body weight trajectory, and withdrawal analyses. Then, offspring of each sex were randomized as follows: three for plasma EOPs and hormones, two for nociception and pharmacokinetics, and two for radiotelemetry.

### Jugular vein catheterization surgery

Twelve female rats were anesthetized (1.5–3.5% isoflurane in oxygen) and placed on a thermoregulated surgical table; the hair covering the right jugular vein was shaved and sterilized with a povidone solution. A non-steroid anti-inflammatory (carprofen, 6 mg/kg) was administered subcutaneously. A 2-cm incision in the scapular region and a 1 cm incision, about 2 cm to the right of the midline, were made. A magnetic vascular access backport (VABR1B/22, Instech Laboratories, Inc., Plymouth Meeting, PA) was inserted through the scapular region, fitted with a 14 cm 22-gauge catheter with a rounded tip and a stopper at 2.5 cm (C30PU-RJV1301, Instech). The jugular vein was isolated, and 2.5 cm of the catheter was inserted and secured. The backport was secured and flushed using a heparinized solution (1% heparin in sterile 0.9% NaCl). A protective magnetic aluminum cap (VABRC, Instech) protected the access button. Following catheterization surgery, rats were allowed a five-day recovery period and were flushed with the heparinized solution daily.

### Fentanyl self-administration exposure model

Operant conditioning chambers (Med Associates, Inc.) were contained in sound-attenuating cabinets with two levers on the right wall, a cue light placed above each lever, and a house light at the top of the chamber between the two levers. Syringe pumps, placed outside the chamber but inside the sound-isolating cabinet, were connected to a free-moving swivel that attached to the backport of the animal through a steel spring-encased tether (KVABR1T/22, Instech). FEN citrate (Fresenius Kabi, LLC., Henry Schein, 1,401,539 using DEA registration number RL0457730) was prepared in physiological sterile saline and delivered intravenously via vascular access backport [32,33].

For lever press training, rats were exposed to a 1 h FEN autoshaping protocol (non-contingent FEN paired with lever presentation) followed by a 2 h operant conditioning session (FEN infusion contingent on lever pressing on a FR1 schedule to train the dams for self-administration [34,35]. After seven days of such training (pre-conceptional exposure), female rats were paired with males during the proestrus phase for breeding. This was followed by 21 days of FEN-SA (2.5 μg/kg/infusion) where both active and inactive levers were available in 2 h daily sessions, on an FR1 schedule of reinforcement. Each active lever press triggered an infusion of FEN, illumination of cue lights, and a 20 s time-out period signaled by switched-off house light. During the time-out period, active lever presses were recorded but did not result in FEN infusions. Inactive lever presses were without programmed consequences at all times. CTL rats received vehicle self-administration (VEH-SA) infusions (0.9% NaCl) in both the autoshaping and self-administration sessions. FEN consumption (Figure 1B) was associated with discriminability between levers higher than 66.6% in FEN-SA dams (Figure 1C), while pregnancy weight gain and duration were similar between groups (Figure 1D). Discriminability higher than 66.6% (2:1 ratio) determined the ability of the rats to distinguish between active and inactive levers, reflecting their understanding of which action leads to drug delivery. Of the 12 dams, 2 in each group did not become pregnant or lost pregnancy, resulting in a final total of 4 litters in the VEH-SA group and 4 litters in the FEN-SA group.

## Morphometrics analysis of the pups

During the first 8 h of life, morphometric measures were assessed as previously reported [36]. Pups were placed on a heating pad. They were weighed, and full length (nose to last caudal vertebra), tail length (anus to last caudal vertebra), body length (nose to anus), and anogenital distance were measured using a caliper. Head and waist circumference were measured using a silk thread. The cephalization index was calculated as the ratio of head circumference to body weight.

### Withdrawal somatic signs analysis

To assess the withdrawal somatic signs in the offspring, 10 min videos were recorded on PD0, 1, and 3 and analyzed as previously described [37,38]. Only the first 10 s of each 30 s segment were scored, thus resulting in a total of 20 (10 s) segments per video to be scored. The presence of each somatic sign was scored as ‘1’ and its absence as ‘0’ for each sign in every 10 s segment and averaged for each offspring. Video scoring, blinded to pup sex and treatment, rated the frequency of head movements, body curls, foreleg movements, hindleg movements, and locomotion. These frequencies were summed to determine a global withdrawal score (GWS). Spontaneous myoclonic jerks (SMJs) and tremors were reported separately.

### Endogenous opioid peptides and adrenal-derived hormones

Plasma met-enkephalin, β-endorphin, and dynorphin-A (S-1419, S1264, S1203, BMA, Biomedicals, Augst, Switzerland, respectively), norepinephrine and metanephrine (BAE 5200 Rand BAE-8100R, respectively, Rocky Mountain Diagnostics), adrenocorticotrophic hormone (ACTH, EK-001–21, Phoenix Pharmaceutical, Burlingame, CA), and corticosterone and aldosterone (Cat. 501,320 and Cat. 501090, respectively, Cayman Chemical Company, Ann Arbor, MI) were extracted and measured by ELISA following the manufacturer’s protocols.

### Plasma fentanyl systemic exposure

At PD120, a blood sample (~250 μl) was collected at baseline (T0) via the tail vein (*n*=6). The animals were then injected with an analgesic dose of FEN (20 µg/kg s.c.), and a blood sample (~250 µl) was collected at 15, 30, and 60 min and 6, 12, and 24 h in EDTA tubes. Blood was centrifuged, and plasma was separated and saved at −80°C. Plasma concentrations of FEN and the primary metabolite norfentanyl (NOR) were quantified using published methods [39–41]. Briefly, plasma samples were extracted via precipitation with ice-cold LC-MS grade methanol spiked with stable deuterium-labeled internal standard (FEN-d_5_ and NOR-d_5_). Cleared extracts were analyzed using LC-MS/MS using a Sciex QTRAP 6500+ coupled to an ExionLC AC high-performance liquid chromatography system. Analytes were separated using a Waters Acquity BEH C18 (1.7 µm; 2.1 × 100 mm) column using a mobile phase composition of (a) 0.1% formic acid in water and (b) 0.1% formic acid in acetonitrile and gradient elution as previously described. Analytes were detected using positive electrospray ionization with the following transitions: 337.1>188.1 for FEN, 233.1>84.0 for NOR, 341.1>188.1 for FEN-d_5_, and 238.0>84.0 for NOR-d_5_. Plasma FEN and NOR concentrations were quantified using the peak area ratios of FEN/FEN-d_5_ or NOR/NOR-d_5_ measured against calibration curves prepared using plasma spiked with known drug concentrations. Spiked quality control (QC) samples at four concentrations were used to validate quantification analysis, and 75–100% of all QC replicates at each level were within 5–10% of the expected value [39–41].

### Hot plate test

All nociception assays were completed by the University of Kentucky Rodent Behavior Core (RBC) in a blinded manner. On the day of the experiment, PD21 or PD120 rats (*n*=6 per group and sex) were placed in individual Plexiglas chambers on an elevated glass surface maintained at 52°C. A radiant heat source was focused on the plantar surface of the hind paw, and the latency to paw withdrawal was recorded by an examiner blinded to group assignment. Each trial was repeated five times at 5-min intervals, and the mean latency was calculated for analysis. The baseline response was recorded in all rats. A week later, rats were given a single dose of FEN citrate (20 μg/kg, s.c.). After 10–15 min, the latency of paw withdrawal was assessed once and repeated every 60 min for 5 consecutive hours.

### Blood pressure and heart rate measurement

At PD90, female littermates (*n*=6 per group) were implanted with radiotelemeters in the descending aorta (HD-10, Data Sciences, Inc., St. Paul, MN) for blood pressure and HR measurements as previously reported [42,43]. Carprofen (10 mg/kg, s.c.) was injected before the surgery, 12 h post-surgery, and once a day for the next three consecutive days. Blood pressure and HR were monitored continuously in freely moving, conscious animals using the Ponemah 6.12 software. A baseline mean arterial pressure (MAP) and HR were collected during a 3:00 PM to 6:00 PM window. Then, a FEN citrate injection (20, 200 μg/kg, s.c.) was administered, and rats were monitored until full recovery.

### Statistical analysis

All data are presented as mean ± SEM. Data for FEN-SA and body composition trajectories were analyzed using repeated-measures (RMs) two-way ANOVA followed by Tukey’s *post-hoc* test. Single-point comparisons for plasma markers, blood pressure, or behavior were analyzed by t-test or two-way ANOVAs.

For somatic signs of withdrawal, the behavioral variables (i.e., GWS, SMJ, and tremors) obtained on PDs 0, 1, and 3 were separately analyzed using RM ANOVAs (between-subjects factor: treatment), with separate analyses conducted for each sex. SMJ was not measured at P0; therefore, the corresponding ANOVA only included P1 and 3 as within-measures. The type I error rate was set at *α*<0.05.

## Results

### Fentanyl acquisition and self-administration

All dams used for this study acquired FEN-SA. FEN intake increased during the 2 h contingent delivery sessions following the 1 h non-contingent auto-shaping period and continued to increase through mating and subsequent 2 h contingent FEN sessions. A notable decrease in FEN intake was observed at gestational day 2 (GD2), after which intake remained relatively stable for the remainder of self-administration training (Figure 1B). Intake changes were likely unrelated to learning of the lever-pressing task as discriminability between active and inactive levers remained fairly consistent throughout the training in FEN-SA rats, while VEH-SA rats showed larger day-to-day fluctuations (Figure 1C). Both FEN-SA and VEH-SA rats exhibited similar pregnancy durations and patterns of weight gain from pre-conception to delivery (Figure 1D).

### Morphometric measurements in neonates

**Figure 2:**
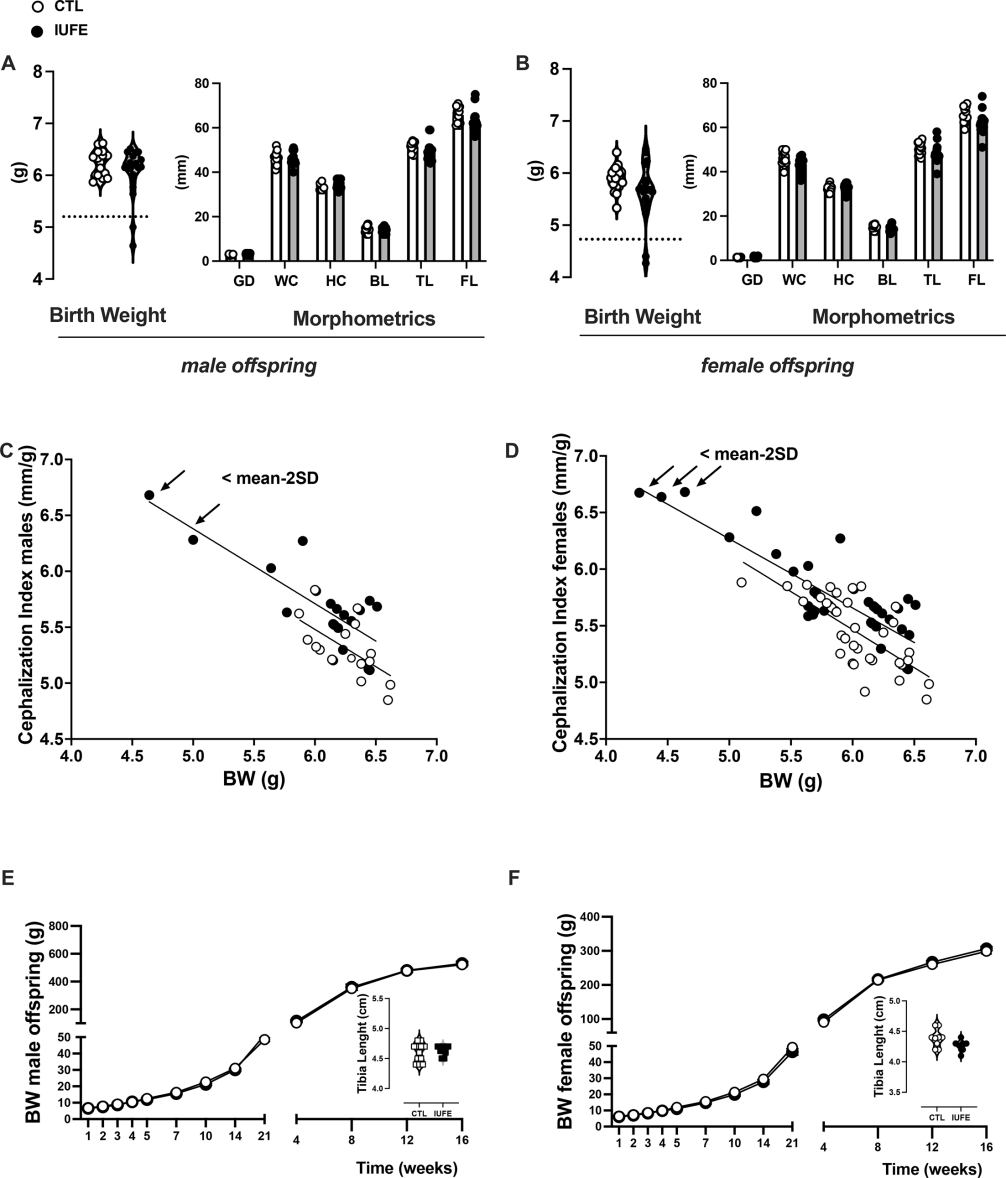
Pregnancy outcomes in dams self-administering fentanyl or vehicle during pre-conception and pregnancy (**A**) birth weight and morphometric analysis in male offspring, (**B**) birth weight and morphometric analysis in female offspring, (**C**) cephalization index and body weight correlation in male offspring, (**D**) cephalization index and body weight correlation in female offspring, (**E**) body weight gain trajectory and tibia length in adult male offspring, and (**F**) body weight gain trajectory and tibia length in adult female offspring. Data were collected, analyzed, averaged per group, and reported as mean ± SEM; *n*=22 males and 18 females per group. BL, body length; BW, body weight; CTL, control, offspring from dams self-administering saline; FL, full body length; GD, anogenital distance; HC, head circumference; IUFE*, in utero* fentanyl exposure, offspring from dams self-administering fentanyl; TL, tail length; WC, waist circumference.

Litter size was not influenced by IUFE (6.5 ± 0.5 CTL males versus 6.2 ± 0.6 IUFE males and 4.2 ± 0.6 CTL females versus 4.7 ± 0.6 IUFE females). Birth weights and morphometric measurements showed no differences in anogenital distance, head circumference, waist circumference, body length, full length, and tail length between CTL and IUFE offspring (Figure 2A–B). Although birth weight and cephalization index showed a negative correlation in both groups (Figure 2C–D), only a small number of IUFE pups in the whole cohort displayed a birth weight below the (mean minus 2 standard deviations, mean-2SD) along with an increased cephalization index. To avoid confounding effects, these pups were removed from the analysis. Importantly, IUFE did not influence weight gain through the course of the study in male and female offspring (Figure 2E–F). Furthermore, tibia length (Figure 2E–F, insets) and organ weights were similar between CTL and IUFE offspring regardless of sex (Supplementary Figure S1).

### Somatic signs of withdrawal

**Figure 3:**
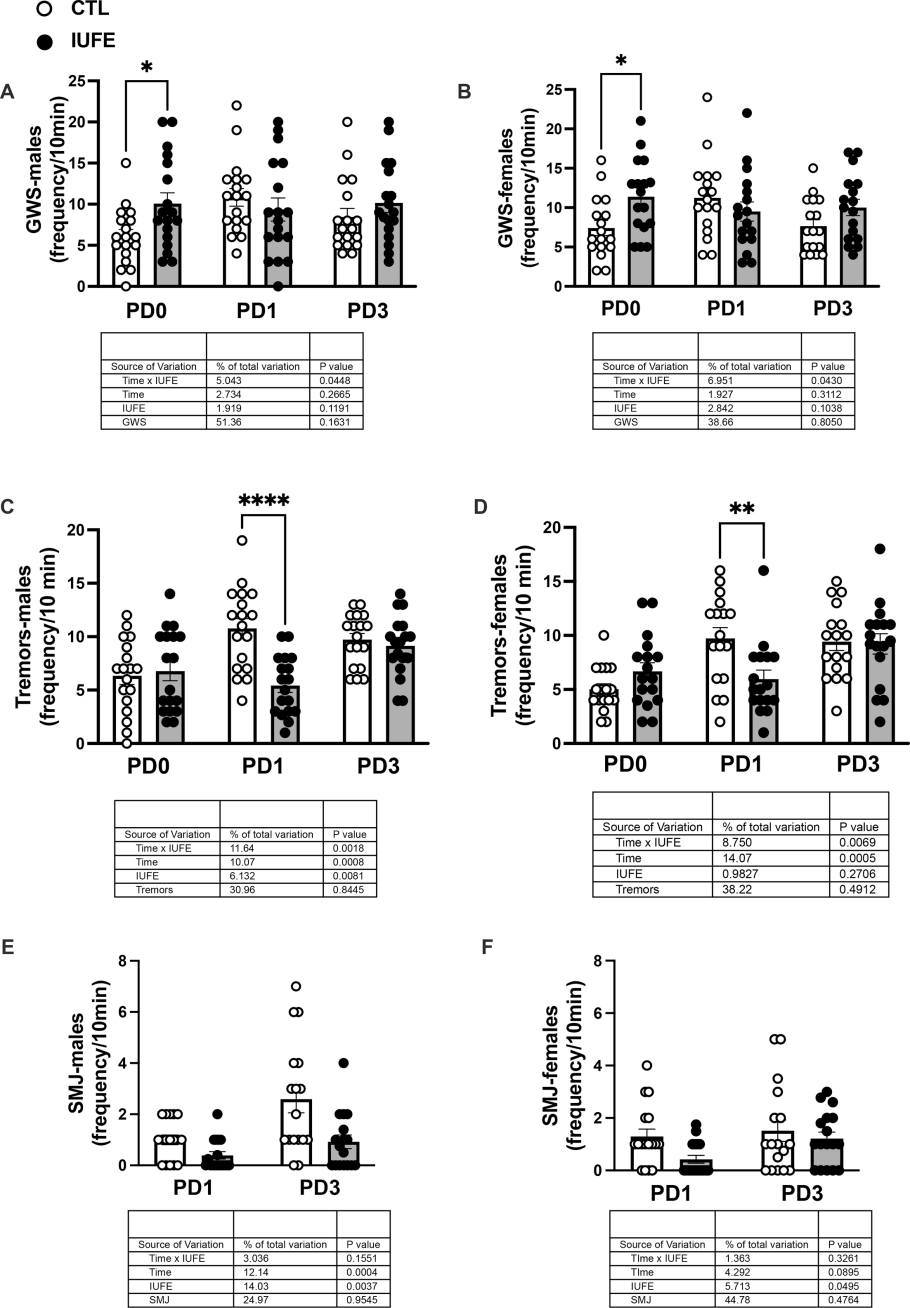
Somatic signs of withdrawal in IUFE and CTL offspring at PD0, 1, and 3 (**A**) GWS in male offspring, (**B**) GWS in female offspring, (**C**) tremors in male offspring, (**D**) tremors in female offspring, (**E**) SMJ-like movements in male offspring, (**F**) SMJ-like movements in female offspring. Data were collected, analyzed, averaged per group, and reported as mean ± SEM; *n*=22 males and 18 females per group. *P<0.05 versus CTL. CTL, control, offspring from dams self-administering saline; GWS, global withdrawal score; IUFE*, in utero* fentanyl exposure, offspring from dams self-administering fentanyl; PD, postnatal day; SMJ, spontaneous myotonic jerks. *P<0.05 versus control.

The RM ANOVAs for the GWS indicated a significant interaction between treatment (IUFE) and PD0, 1, 3 of measurement (F_2.51_=3.30, *P*=0.045 and F_2.48_=3.36, *P*=0.043, respectively) for both male and female offspring. Tukey’s *post-hoc* tests indicated that IUFE increased GWS at PD0, but not at PD1 and PD3 (Figure 3A–B). 

For tremors, there was a significant interaction between PD of assessment and maternal treatment in male (F_2.51_=7.20, *P*=0.002) and female offspring (F_2.48_=8.84, *P*=0.007). The *post-hoc* tests indicated that IUFE significantly suppressed tremors when compared with the CTL group at PD1 in both sexes (Figure 3C–D). Furthermore, IUFE male and female offspring exhibited significantly less SMJ than CTL counterparts (Figure 3E–F), across PD1-3 (F_1.32_=9.80, *P*=0.004, and F_1.32_=4.17, *P*=0.049, respectively). Within the measured behavioral variables, sex did not exert a significant main effect nor was it involved in significant interactions.

### Plasma opioid peptides and adrenal-derived hormones

**Figure 4:**
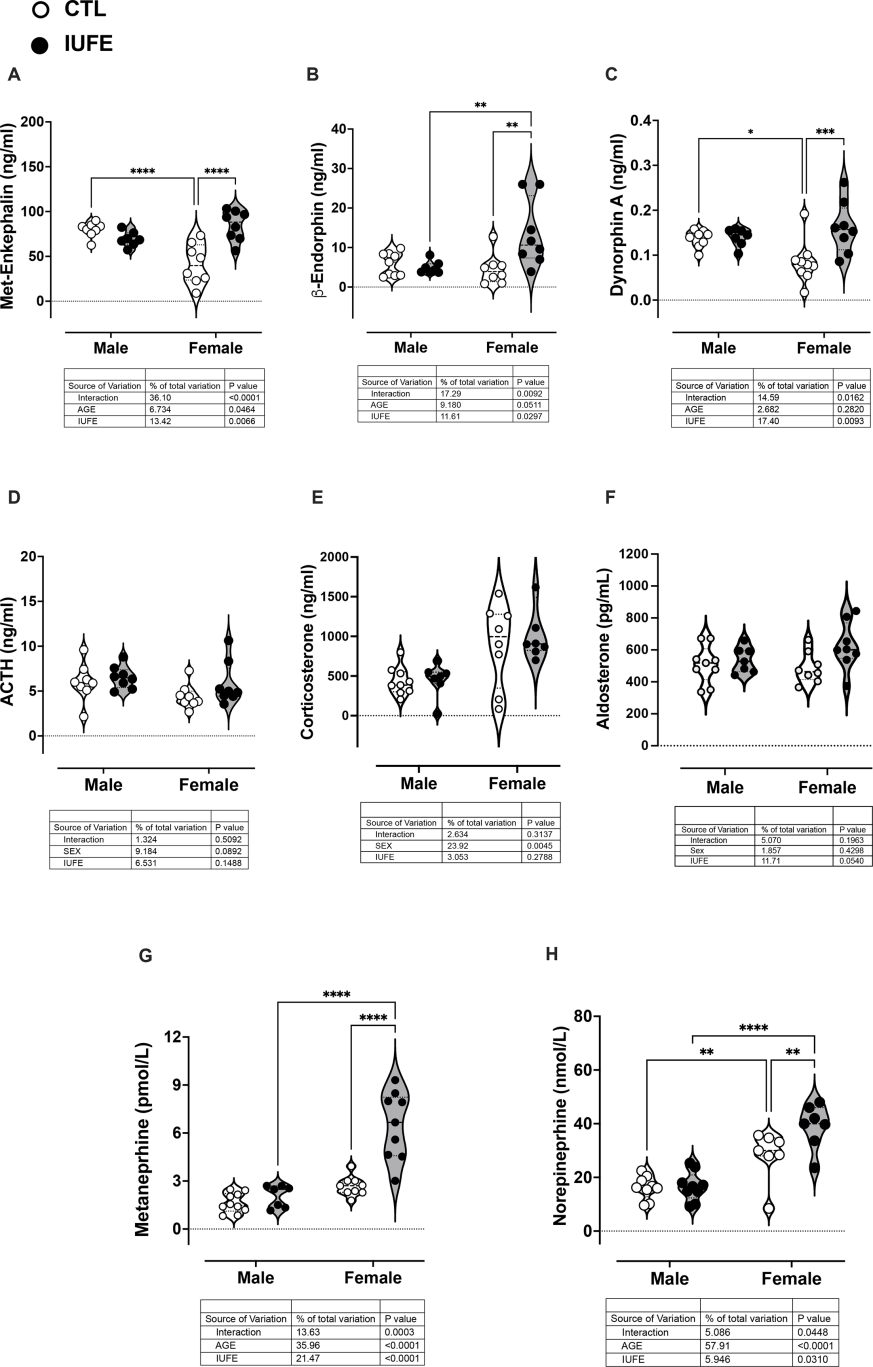
Plasma endogenous opioid peptides and adrenal-derived hormones in adult male and female CTL and IUFE offspring Plasma levels of (**A**) met-enkephalin, (**B**) β-endorphin, (**C**) dynorphin A, (**D**) ACTH, (**E**) corticosterone, (**F**) aldosterone, (**G**) metanephrine, and (**H**) norepinephrine. Data were collected, analyzed, averaged per group, and reported as mean ± SEM; *n*=6 females per group. *P<0.05 versus CTL. µg=microgram; kg=kilogram; g=gram. CTL, control, offspring from dams self-administering saline; FEN, fentanyl; IUFE*, in utero* fentanyl exposure, offspring from dams self-administering fentanyl; SA, self-administration; VEH, vehicle.

Plasma met-enkephalin levels were reduced in weanlings, while dynorphin A, β-endorphin, and adrenal-derived hormone levels were similar between CTL and IUFE weanlings (Table 1). In the adult offspring, only females with IUFE showed increased met-enkephalin, β-endorphin, and dynorphin A levels compared with CTL counterparts (Figure 4A–C).

**Table 1:** 

	CTL-male	IUFE-male	CTL-female	IUFE-female	p^int^	p^sex^	p^IUFE^
Met-ENK (ng/ml)	34.75 ± 6.46	13.86 ± 4.75	43.44 ± 7.86	16.62 ± 5.22	0.644	0.375	0.001
b-endorphin (ng/ml)	0.42 ± 0.04	0.73 ± 0.04	0.48 ± 0.08	0.84 ± 0.14	0.784	0.376	0.001
Dynorphin A (ng/ml)	0.091 ± 0.003	0.075 ± 0.004	0.126 ± 0.014	0.092 ± 0.005	0.303	0.005	0.006
ACTH (ng/ml)	8.7 ± 1.85	7.51 ± 1.12	4.00 ± 0.37	4.75 ± 0.76	0.534	0.067	0.826
Corticosterone (ng/ml)	136.4 ± 23.5	121.0 ± 22.0	139.6 ± 21.3	131.1 ± 17.8	0. 822	0.713	0.549
Aldosterone (ng/ml)	3.20 ± 0.76	2.92 ± 0.42	4.75 ± 0.58	3.90 ± 0.78	0.556	0.142	0.579
Norepinephrine (nmol/l)	54.63 ± 6.49	43.37 ± 3.92	56.75 ± 2.80	54.66 ± 7.84	0.642	0.413	0.147
Metanephrine (nmol/l)	3.35 ± 0.66	11.52 ± 0.85	7.03 ± 0.82	11.07 ± 1.98	0.128	0.190	0.012

Plasma ACTH was not influenced by IUFE in either sex (Figure 4D). Similarly, the adrenal cortex-derived hormones, aldosterone and corticosterone, were similar between groups (Figure 4E–F). However, IUFE increased adrenal medulla-derived hormones, plasma norepinephrine, and metanephrine in weanlings from both sexes and female adult rats compared with CTL (Figure 4G–H).

### Thermal nociception

**Figure 5:**
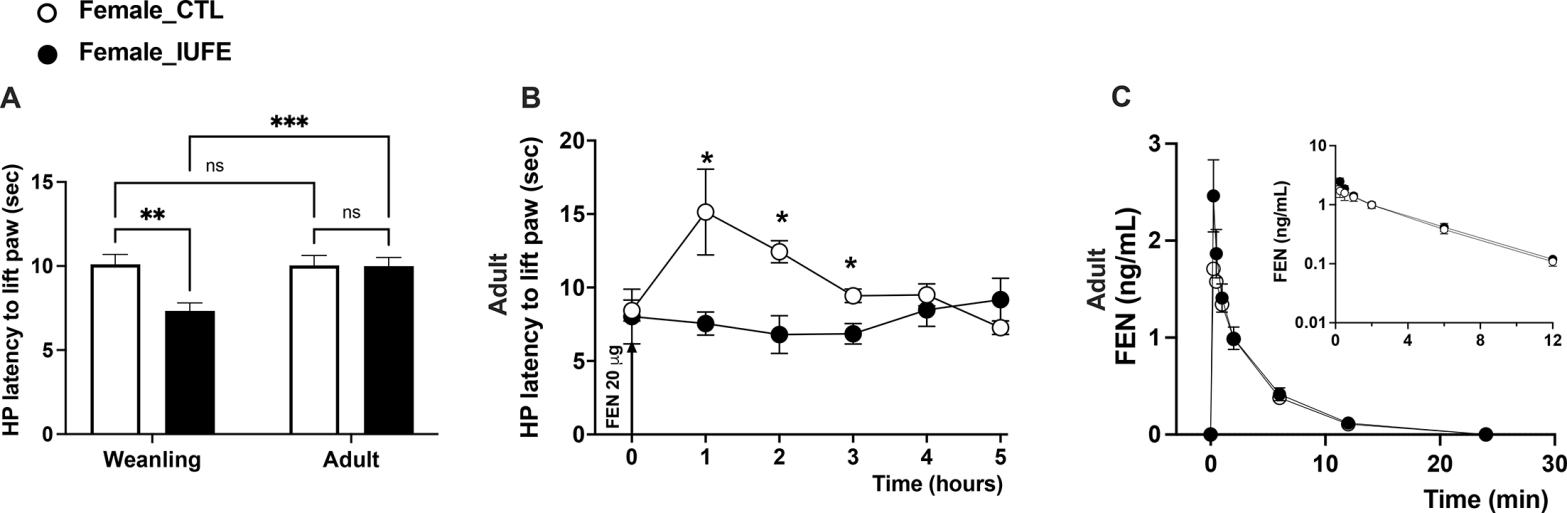
Nociception and plasma fentanyl exposure in adult CTL and IUFE offspring (**A**) hot plate test in weanling and adult female offspring, (**B**) progressive hot plate test in response to a 20 χg/kg dose of fentanyl, (**C**) pharmacokinetics of fentanyl in response to a 20 µg/kg dose, i.p. Data were collected, analyzed, averaged per group, and reported as mean ± SEM; *n*=6 females per group. *P<0.05 versus CTL. CTL, control, offspring from dams self-administering saline; HP, hot plate test; IUFE*, in utero* fentanyl exposure, offspring from dams self-administering fentanyl.

Latency for paw withdrawal in response to heat was reduced in female IUFE weanlings compared with female CTL counterparts; however, this response was similar between these two groups later in life (Figure 5A). Nociception was further tested in response to a dose of FEN (20 μg/kg, s.c.) in adult female rats. As expected, female CTL offspring showed increased latency for paw withdrawal during the first 3 h after the injection; however, female IUFE offspring displayed FEN-induced hyperalgesia (Figure 5B).

### Plasma fentanyl systemic exposure

The plasma concentration-time profiles for FEN after 20 μg/kg s.c. injection were similar in adult female CTL and IUFE offspring (Figure 5C). FEN plasma concentrations in adult rats with IUFE were similar to their CTL counterparts. At 15 min, the average concentration of FEN in the CTL group was 1.71 ± 0.38 ng/ml (95% CI: 0.75–2.68 ng/ml) compared with 2.46 ± 0.37 ng/ml (95% CI: 1.51–3.42 ng/ml) in the IUFE offspring. Concentrations of FEN declined mono-exponentially at similar rates in CTL and IUFE offspring (Figure 5C, inset). At 24 h, plasma FEN was below the lower limit of quantification or was undetected in the plasma for all animals. The concentrations of the primary inactive metabolite, NOR, were near the limit of quantification, and all time points were below FEN plasma concentrations. The appearance of NOR in plasma occurred between 15 min and 1 h, was highly variable, and was undetectable after 12 h in all animals (not shown). IUFE did not influence FEN pharmacokinetics in male offspring either (Supplementary Figure S2). Collectively, FEN plasma levels were similar in both groups of rats, suggesting that IUFE does not influence the pharmacokinetics of FEN in adult female and male offspring.

### Pressor response to acute doses of fentanyl

**Figure 6:**
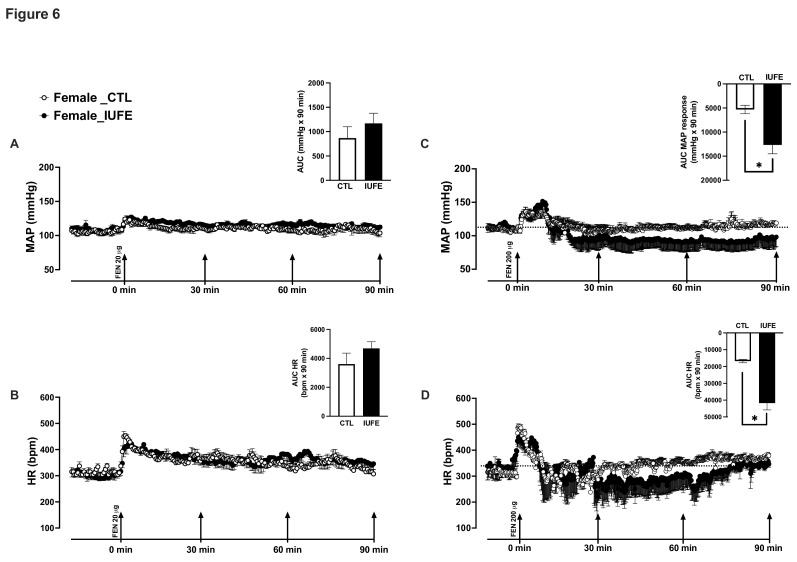
Cardiovascular response of adult female IUFE and CTL offspring to different fentanyl doses (**A**) MAP in response to 20 μg/kg of fentanyl, **(B)** HR in response to 20 μg/kg of fentanyl, (**C**) MAP in response to 200 μg/kg of fentanyl, (**D**) HR in response to 200 μg/kg of fentanyl. Data were collected, analyzed, averaged per group, and reported as mean ± SEM; *n*=6 females per group. *P<0.05 versus CTL. CTL, control, offspring from dams self-administering saline; HR, heart rate; IUFE*, in utero* fentanyl exposure, offspring from dams self-administering fentanyl; MAP, mean arterial pressure.

Adult female rats were implanted with radiotelemetry and subjected to a low analgesic dose of FEN (20 μg/kg, s.c.) or a high analgesic dose of FEN (200 μg/kg, s.c.). At the lower dose, changes in MAP and HR were similar between CTL and IUFE rats (Figure 6A,B), as denoted by similar areas under the curve. However, the higher dose of FEN induced a catatonic state associated with a reduction in MAP and HR in both groups; however, this response was exacerbated in rats with IUFE compared with CTL rats (Figure 6C,D).

## Discussion

This is the first study to report that a model of maternal FEN-SA dysregulates EOPs and increases the metanephrine levels in male and female weanlings with IUFE. These neuroendocrine alterations at PD21 were preceded by increased somatic signs of withdrawal in early postnatal life, despite the absence of obvious birth complications or morphometric alterations. Nevertheless, only adult female offspring with IUFE showed long-lasting dysregulation of EOPs and adrenal-derived hormones. Specifically, our data shows that maternal use of FEN during preconception and gestational periods may be associated with the chronic activation of the sympathoadrenal-medullary axis in female offspring, which also showed FEN-induced hyperalgesia and a greater FEN-induced hypotensive and bradycardic response. Thus, we have identified potential sex-specific health risks associated with opioid re-exposure in adult offspring with IUFE.

There are several challenges to recapitulating the length and severity of opioid exposure in preclinical models. We used intravenous self-administration, a ‘gold standard’ method, which is associated with constant fluctuations in plasma FEN levels, unlike continuous infusions or subcutaneous experimenter-delivered injections. This is useful to investigate the interaction between the reinforcing mechanisms of substance use disorder as well as other less studied physiological comorbidities, such as metabolic or cardiovascular disease. Our preclinical model involved training for FEN self-administration before and during mating and throughout pregnancy and showed that weight gain, gestation length, and number of pups per litter were similar in female dams self-administering vehicle or FEN. Overall, offspring with IUFE showed similar birth weights to those of their CTL counterparts. However, a small subset (~11%) of IUFE offspring was born smaller than the mean-2SD of the group. Notably, the incidence of low birth weight in the United States is about 8.42%, while maternal OUD doubles this risk [44,45]. Yet, numerous clinical studies highlight the severity of the outcomes in newborns showing prematurity or low birth weight associated with NOWS, creating the general assumption that these two factors are present in all newborns with IUFE. Importantly, after removing offspring with birth weight lower than the mean-2SD from the analysis, we showed that IUFE induced adverse nociceptive and cardiovascular effects in response to opioid re-exposure in the adult offspring, as well as neuroendocrine factors dysregulation. Therefore, our model provides a reliable tool to investigate the mechanisms linking IUFE with greater health risks to opioid re-exposure independent of low birth weight, a marker of intrauterine growth restriction, as a confounding factor.

Neonates with IUFE exhibited behavioral patterns indicative of withdrawal: significantly higher levels of overall activity (body curls, head movements, limb movements, and locomotion) compared with the CTL group, while simultaneously displaying reduced SMJ. The latter movements are part of the natural motor development observed in early postnatal stages are believed to be involuntary muscle contractions that contribute to the maturation of the neuromuscular system [46]. It is also intriguing that the exacerbation in GWS, observed in neonates with IUFE, was greater at P0 compared with P1 or P3. This temporal pattern suggests that heightened arousal and agitation in pups with IUFE decreased over time along with hallmark signs of opioid withdrawal [47]. However, other negative signs persisted, such as a reduced frequency of tremors and SMJ-like movements in IUFE pups. These reductions may have also been present at PD0 but were likely overshadowed by the overall heightened activity associated with opioid exposure. Although tremors are a hallmark sign of opioid withdrawal, their reduced frequency in our model should not be interpreted as a healthy outcome. Instead, pups with IUFE displayed a shift toward other abnormal motor patterns, including heightened head, body, and limb movements. This pattern suggests a qualitative change in the expression of withdrawal rather than an attenuation of symptoms.

IUFE resulted in dysregulation of EOPs in male and female weanlings, as it led to reduced met-enkephalin levels. Reduced levels of met-enkephalin are most likely secondary to potent stimulation of μ−OR with a synthetic agonist during prenatal life as a compensatory mechanism to down-regulate the endogenous receptor activation. Later in life, EOP levels were increased only in adult female offspring. This may reflect complex, long-lasting neurobiological adaptations induced by IUFE, involving multiple components of the opioid system and related neural circuits. These effects could be due to alterations in gene expression, peptide synthesis, receptor regulation, and neurotransmitter dynamics that contribute to altered affective and reward processing in adulthood [48,49]. Such changes likely reflect both neurodevelopmental remodeling due to early developmental opioid exposure and compensatory adaptations to chronic opioid disruption, resulting in persistent opioid system dysregulation. For example, dynorphins may increase to counterbalance the chronic activation of μ−OR by inhibiting dopamine release via κ-OR, similar to what has been reported in models of prenatal alcohol exposure [50]. This neurobiological state of kappa system activation is associated with heightened dysphoria that can promote drug use as a coping mechanism for negative affective states. For instance, Lepreux et al. found that overexpression of the gene coding for kappa receptors facilitates alcohol self-administration and depressive-like behavior [51]. Conversely, Wille-Bille et al. observed alterations in opioid gene expression after prenatal ethanol exposure [52]. Taken together, these findings suggest that IUFE results in a withdrawal-like state and a dysregulated opioid system, potentially increasing vulnerability to substance use later in life.

Although increases in β-endorphins are a physiological response in newborns, the levels rapidly decline within a week. However, prenatal exposure to maternal opioid misuse increases the neonatal plasma endorphins for several months after birth [26–28]. In our model, female offspring display long-lasting effects that could respond to hormonal [53] or epigenetic [54] changes in the production and release of EOPs. Particularly relevant to this study, drug-induced transgenerational epigenetic inheritance has been associated with increased anxiety-like behavior during adolescence and increased sensitivity to the analgesic effects of morphine [55]. Moreover, maternal consumption of an obesogenic diet can induce epigenetic changes (DNA hypomethylation) in their offspring, associated with long-term alterations in the expression of opioid and dopamine peptides and the preference for palatable foods [56]. Nevertheless, the sex-specific nature of the long-term alterations in the plasma profile of EOPs, as well as the changes in the temporo-spatial expression of ORs, warrants further investigation.

NOWS is a strong predictor of attention-deficit hyperactivity disorder in children exposed to opioids, compared with unexposed CTLs matched for relevant variables [57–59]. Despite their variability in the diagnosis and treatment of NOWS, retrospective chart reviews and medical record evaluations offer the only source for addressing potential problems concerning the long-term consequences of IUFE [60]. These sources suggest that attention-deficit hyperactivity disorder may reflect atypical activation of the locus coeruleus–norepinephrine (LC–NE) system [61]. The LC–NE system is known to play a key role in activating the sympatho-adrenomedullary (SAM) axis, which mobilizes energy stores, increases HR and blood pressure, and prepares the body for ‘fight or flight’ responses [62,63]. Our study shows that female offspring with IUFE undergo the chronic activation of the SAM system, as they exhibit increased levels of norepinephrine and epinephrine, metabolized to metanephrine by the enzyme catechol-O-methyltransferase. This process occurs extraneuronally, in the adrenal medulla and in the paraganglia of the sympathetic nervous system [64]. Notably, withdrawal from misused substances, like alcohol, benzodiazepines, and opioids, as well as stress and exercise can increase the levels of metanephrine [65]. This finding will guide future follow-up studies in children with IUFE to achieve an early detection of potential neuroendocrine health issues, particularly in females. Of note, our preclinical model points to the chronic activation of the LC–NE system as possible link between IUFE and the increased risk of hyperalgesia and hypotension in the adult offspring.

FEN exhibits the highest affinity for μ−OR, with a strong β-arrestin-biased signaling [8]. This selectivity profile explains the predominance of μ−OR-mediated effects, such as analgesia, euphoria, and respiratory depression, with severe cases involving inhibition of the hypoxic and hypercapnic ventilatory responses and even death with overdose [66–69]. The choice for pain medication is based on both the analgesic properties of a drug and the side effects on the critical control of arterial blood pressure [70,71]. Opioid drugs bind to μ−ORs in spinal and brainstem regions involved in pain but also in regions regulating breathing [72,73]. Activation of opioid receptors in the central nervous system (e.g. nucleus ambiguous) evokes bradycardia, which is mediated by increases in inhibitory parasympathetic activity to the heart [74]. Because the prehospital setting shows limitations in access to cardiovascular support, avoiding hypotension is a major priority to prevent cardiorespiratory depression [75]. Currently, opioid-based medications used in prehospital settings to treat pain include FEN, morphine, sufentanil, and ketamine (N-methyl-D-aspartate receptors antagonists). However, all these medications drastically affect autonomic cardiovascular regulation in conscious humans [76], as well as anesthetized humans [77,78] and anesthetized animal models [79,80]. Our current data support the notion that the acute FEN administration to adult offspring in similar analgesic doses as those used in the prehospital setting reduces pain perception in CTL rats but not in IUFE counterparts. This response could be mediated by central dysregulation of the EOPs since the hot plate test shows the supraspinal integration of nociceptive responses. Opioid-induced bradycardia and hypotension can be clinically significant, especially in susceptible patients with underlying cardiac conditions. Overall, literature addressing the effect of FEN misuse on cardiovascular and metabolic disease is scarce, and there is a dramatic gap in reports concerning offspring with prenatal exposure to opioids. Thus, our study lends weight to the idea that IUFE may lead to a higher risk of cardiorespiratory depression associated with FEN re-exposure. Future studies will determine whether targeting EOPs centrally and peripherally may serve as an effective approach to reduce health risks in adult offspring with IUFE.

Another finding of our study uncovers that IUFE and CTL animals display similar plasma drug exposure after an acute dose of FEN. Therefore, the differences we observe in algesia, hypotension, and bradycardia are not due to the effects of IUFE on FEN plasma pharmacokinetics. Our data are the first to demonstrate that FEN re-exposure induced hyperalgesia and exacerbated hypotension and bradycardia in adult offspring with IUFE. Thus, the results from this work may provide insights into future guidelines for the use of FEN in both outpatient and hospital settings in people with IUFE.

A limitation of this study is the opportunity to assess algesia and cardiovascular function using radiotelemetry only in adult female offspring. Based on the plasma profile, males do not show long-term alterations in EOPs and SAM activation; however, differences in the physiological response to FEN re-exposure remain to be determined before concluding that IUFE induces a sex-specific effect in nociceptive and blood pressure control. Further, a new novel syndrome has been described in the literature for prenatal FEN exposure, characterized by short stature, microcephaly, distinctive facial features, and congenital malformations [81]. This is not associated with genetic or genomic causes, aside from their nonprescription exposure to FEN during pregnancy [81]. Our study shows that only a small number of offspring manifest signs of brain vulnerability, such as increased cephalic index. Thus, prospective studies could attempt to train female rats to self-administer higher doses of FEN using the escalation paradigm to investigate the impact on the neurodevelopment of the offspring.

In conclusion, we modeled maternal OUD by subjecting female rats to self-administration during preconception and gestation. Male and female offspring displayed somatic signs of withdrawal along with circulating EOPs dysregulation; however, increased EOPs and adrenal-derived hormones associated with FEN-induced hyperalgesia and exacerbated cardioinhibitory response to high analgesic doses of FEN were found only in adult female offspring. This model not only advances our understanding of the mechanisms linking IUFE to adverse health outcomes, but also establishes a clinically relevant foundation for developing and testing targeted therapeutic approaches to improve long-term outcomes in maternal-fetal medicine, particularly within the context of OUD.

**Clinical perspectives**

- The limited number of prospective studies in children with prenatal fentanyl exposure raises significant concerns about the lack of knowledge on future comorbidity risks in this population. To halt the progression of the opioid crisis, there is an urgent need to develop translational animal models that investigate opioid use disorder-related health issues in vulnerable populations such as pregnant women and their newborns.
- This is the first study to report that a model of maternal fentanyl self-administration during preconception and full gestation dysregulates the levels of endogenous opioid peptides and activates the sympathomedullary adrenal axis, identifying potential underlying mechanisms for increased health risks associated with opioid re-exposure in adult offspring with prenatal fentanyl exposure.
- There are limited studies examining the chronic effects of prenatal opioid exposure beyond neonatal withdrawal. We provided an unprecedented level of detail on how prenatal opioid exposure negatively influences algesia and blood pressure regulation in the adult offspring. Moreover, our findings highlight the need to limit the use of fentanyl in individuals with known or suspected prenatal opioid exposure, particularly in acute care settings where cardiorespiratory stability is critical.
- Investigating whether prenatal fentanyl exposure is associated with sex-specific differences in the prevalence and severity of non-psychiatric comorbidities—such as metabolic and cardiovascular dysfunctions—will help identify individuals at higher risk and inform targeted intervention strategies.

## Supplementary material

Online supplementary material 1

## Data Availability

The data that support the findings of this study are available from the corresponding author upon reasonable request.

## Open Access

This article has been published open access under our Subscribe to Open programme, made possible through the support of our subscribing institutions, learn more here: https://portlandpress.com/pages/open_access_options_and_prices#conditional

## Abbreviations

CTL: compared with control
EOP: endogenous opioid peptide
FEN: fentanyl
FEN-SA: fentanyl self-administration
FR1: fixed-ratio 1
GWS: global withdrawal score
HPA: hypothalamic–pituitary–adrenal
HR: heart rate
IUFE: *in utero* fentanyl exposure
LC–NE: locus coeruleus–norepinephrine
MAP: mean arterial pressure
NOR: norfentanyl
NOWS: neonatal opioid withdrawal syndrome
OUD: opioid use disorder
PD: postnatal day
QC: quality control
RM: repeated-measure
SAM: sympatho-adrenomedullary
SMJ: spontaneous myoclonic jerk
VEH-SA: vehicle self-administration infusion
κ-OR: kappa OR
μ-OR: mu-opioid receptor
