# Supplementary material for: Fentanyl exposure during preconception and gestation permanently dysregulates endogenous opioid peptides and sympathoadrenal-medullary axis in the offspring
Source: Clin Sci (Lond). 2025 Nov 6;139(21):1337–53. doi: 10.1042/CS20256962 (PMC12751060; doi:10.1042/CS20256962)
Supplement: Online supplementary material 1 [file CS-139-21-CS20256962-s001.docx]

**SUPPLEMENTAL MATERIAL**

**Preconception and gestational fentanyl exposure permanently dysregulates opioid peptides and sympathoadrenal-medullary axis**

^1^Nermin S. Ahmed, ^1^Carolina Dalmasso, ^2^Navid S. Tavakoli, ^5^Pedro Peñalver Abed, ^1^Meghan B. Turner, ^3^Lindsay C. Czuba, ^5^Ricardo M. Pautassi, ^2^Pavel I. Ortinski, ^1,4^Analia S. Loria

1 Department of Pharmacology and Nutritional Sciences, College of Medicine

2 Department of Neuroscience, College of Medicine

3 Department of Pharmaceutical Sciences, College of Pharmacy

4 SAHA Cardiovascular Center

University of Kentucky

Lexington, KY 40536

5 Instituto de Investigaciones Médicas M. y M. Ferreyra (INIMEC-CONICET-UNC) Universidad Nacional de Córdoba, Argentina
